# Subacromial pigmented villonodular synovitis: case report and review

**DOI:** 10.1093/jscr/rjab019

**Published:** 2021-03-08

**Authors:** Vitor Luis Pereira, Arthur Rodrigues Baldan, Carlos Vicente Andreoli, Paulo Santoro Belangero, Alberto de Castro Pochini, Benno Ejnisman

**Affiliations:** Traumatology Sports Center (CETE), (DOT-UNIFESP/EPM), Orthopedics and Traumatology Department of the Escola Paulista de Medicina, Federal University of São Paulo São Paulo, SP, Brazil; Traumatology Sports Center (CETE), (DOT-UNIFESP/EPM), Orthopedics and Traumatology Department of the Escola Paulista de Medicina, Federal University of São Paulo São Paulo, SP, Brazil; Traumatology Sports Center (CETE), (DOT-UNIFESP/EPM), Orthopedics and Traumatology Department of the Escola Paulista de Medicina, Federal University of São Paulo São Paulo, SP, Brazil; Traumatology Sports Center (CETE), (DOT-UNIFESP/EPM), Orthopedics and Traumatology Department of the Escola Paulista de Medicina, Federal University of São Paulo São Paulo, SP, Brazil; Traumatology Sports Center (CETE), (DOT-UNIFESP/EPM), Orthopedics and Traumatology Department of the Escola Paulista de Medicina, Federal University of São Paulo São Paulo, SP, Brazil; Traumatology Sports Center (CETE), (DOT-UNIFESP/EPM), Orthopedics and Traumatology Department of the Escola Paulista de Medicina, Federal University of São Paulo São Paulo, SP, Brazil

## Abstract

Pigmented villonodular synovitis (PVNS) is a non-neoplastic proliferative process that involves synovial tissue in the joints, tendon sheaths and bursae. It usually occurs in young adults, aged 20–50 years, is characteristically monoarticular and of slow progression. Clinical symptoms are nonspecific, and joint stiffness and pain are common in long-term cases. Shoulder PVNS is known to be extremely rare, especially when affecting the subacromial bursal region without joint involvement.

Magnetic resonance is the imaging modality of choice in PVNS and is useful for diagnosis, surgical planning and monitoring. Complete surgical synovectomy remains the treatment of choice, and all pathological synovial tissue should be removed. Patients who are not properly treated can progress to joint destruction.

We describe a case of PVNS in the subacromial bursa of a 15-year-old patient, with exuberant symptoms and 2 years of evolution, treated with extensive synovectomy and good clinical results in the 1-year follow-up.

## INTRODUCTION

Pigmented villonodular synovitis (PVNS) is a non-neoplastic proliferative process that involves synovial tissue in the joints, tendon sheaths and bursae. The first report was made by Chassaignac in 1852 and its definition made by Jaffe and colleagues in 1941 [1, 2]. It is an unusual clinical entity, with an estimated prevalence of 1.8: 1000 000 people. It usually occurs in young adults, aged 20–50 years, with men and women equally affected [2–4].

The origin of such an abnormality is probably inflammatory, and synovial lesions are classified into diffuse and localized forms [2]. The disease is monoarticular and generally of slow progression. Often 2–3 years pass before the patient seeks medical advice. Clinical symptoms are nonspecific, and pain, limited movement, swelling, heat and joint sensitivity are the most frequent complaints [4, 5].

We present a case report of PVNS affecting the bursal subacromial space in the shoulder of an adolescent, without intra-articular disease. It was surgically treated after 2 years of evolution with total arthroscopic synovectomy. Both the patient and her responsible family members agreed to grant consent for the publication of this case report.

## CASE REPORT

Female patient, 15 years old, white, born and raised in São Paulo, sought orthopedic service complaining of pain in her right shoulder 2 years ago. It evolved over the months with worsening pain and a progressive increase in shoulder volume. She sought medical attention during this period; there was no definite diagnosis, and the treatment was based on pain relief, anti-inflammatories and physical therapy. She had worsened pain and increased shoulder volume in the last 6 months. She denied a similar family history, use of chronic medications or knowledge of any comorbidity.

**Figure 1:**
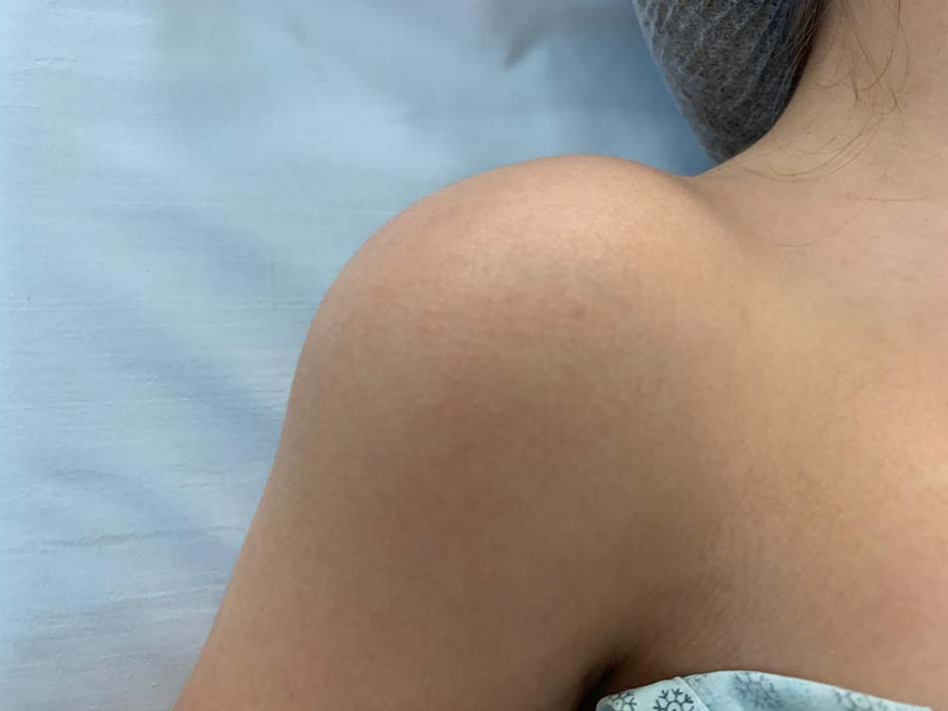
Clinical image of the patient’s right shoulder. We observed an increased volume of the shoulder as a whole, with no changes in the associated skin.

Upon physical examination, we identified a diffuse increased volume throughout the right shoulder, a globally decrease in range of motion (especially in elevation) and pain during passive mobilization of the right upper limb (Fig. 1).

**Figure 2:**
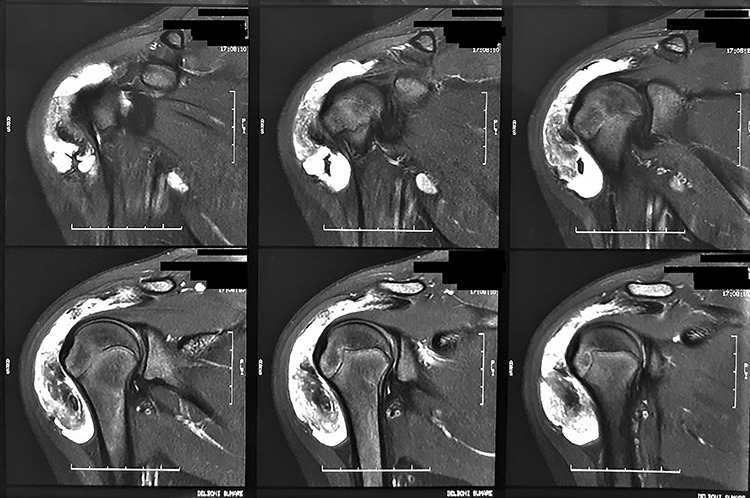
Magnetic resonance images of the patient. We observed coronal sections of the right shoulder in a sensitive liquid sequence, showing intense diffuse subacromial synovial thickening, associated with grouped images in the topography of the bursa.

Radiographs were requested, which did not demonstrate any significant findings. For better diagnostic clarification, a nuclear magnetic resonance imaging of the right shoulder was requested, which showed diffuse synovial thickening and abundant bursal fluid distension without intra-articular involvement (Fig. 2).

**Figure 3:**
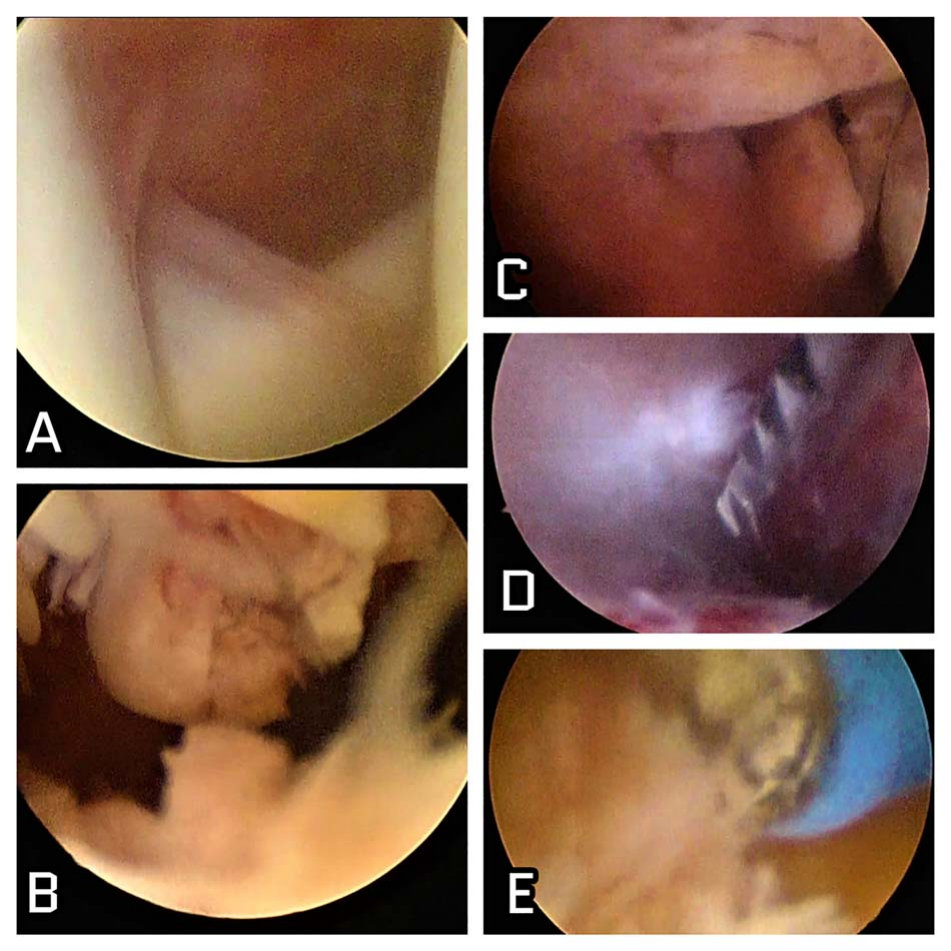
Intraoperative images of the patient. (**A**) Intra-articular image of the shoulder showing absence of local involvement; (**B**) subacromial view showing intense characteristical synovitis; (**C**) nodulations with synovial aspect in the subdeltoid lateral space; (**D**) shaver synovectomy; (**E**) radiofrequency thermal ablation synovectomy as effective arthroscopic resources in surgical treatment.

The patient was treated surgically in an arthroscopic manner with total excision of the synovium, fluid and free bodies, which were sent to pathological anatomy. The intraoperative findings are highlighted in Figure 3. The anatomopathological result was PVNS, confirming the diagnosis.

**Figure 4:**
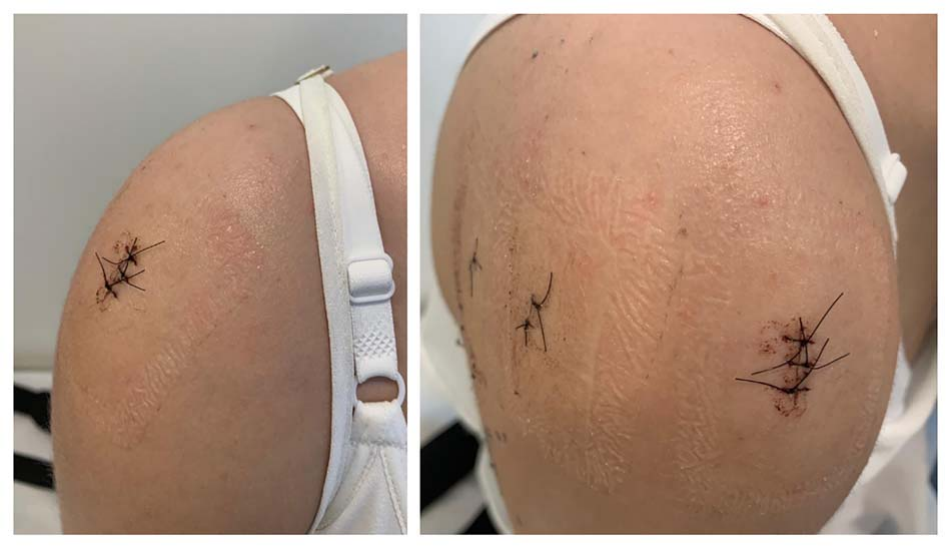
Clinical images of the patient 10 days after surgery. In the left image, we observe an important decrease in the shoulder volume when compared with the preoperative image (Fig. 1). In the right image, we can see the positioning of the arthroscopic portals performed during surgery.

We kept a sling for analgesia by 1 week after surgery. Shoulder stitches were removed after 10 days, and the patient was referred for physical therapy (Fig. 4). She progressed with significant pain improvement and rapid gain in range of motion in the first month.

Three months after the operation, the patient had a normal shoulder range of motion and motor strength was restored. A new ultrasonography of the right shoulder showed the absence of an inflammatory process in the fourth postoperative month. After 1 year of clinical follow-up, the patient maintained functional improvement and completed return to her activities of daily living.

## DISCUSSION

Shoulder PVNS is known to be extremely rare [4]. We found in the literature a total of 33 studies involving <40 case reports associated with shoulder PVNS. However, only three cases were associated with the occurrence of the disease in minors, one of them in a child [6] and two others in adolescents [3, 7]. We did not find any reports specifying exclusive involvement of the subacromial bursa.

PVNS is a proliferation of benign tissue that appears in a borderline between a reactive and a neoplastic process, emanating from the tendosynovial layers, the joint capsule and the synovial pouch. Two forms of PVNS can be differentiated macroscopically: the diffuse form, which involves the entire synovium of a joint, and the localized form with isolated tissue masses in the synovium [4]. The patient was characterized as presenting a localized form of the disease, which in a peculiar way affected only the subacromial region.

Initial radiographs can be negative and calcification is not a common feature [8]. In the final stages of the disease, necrosis and arthritic changes can be seen [4]. Magnetic resonance is the imaging modality of choice and is useful for diagnosis, surgical planning and follow-up [3]. It is the best test to differentiate synovial diseases and perform an early diagnosis. Low-signal areas are observed and correspond to the characteristic hemosiderin deposit within the synovium. The nodular appearance is typical and the assessment of the extent of bone involvement is also important [5].

Differential diagnosis of PVNS must be made with diseases involving joint effusion and synovial thickening and impairment of the joint and neighboring structures such as rheumatoid arthritis, tuberculosis, synovial hemangioma, synovial osteochondromatosis, synovial arborescent lipoma and synovial sarcoma [5]. In the case described, synovial osteochondromatosis was considered as a first hypothesis, until the more accurate arthroscopic characterization of the lesion pointed to PVNS.

Untreated PVNS can invade the surrounding soft tissue and joint, leading to destruction. Therefore, early diagnosis and immediate treatment are essential [3]. Therapeutic approaches include total and local synovectomy, arthroplasties and radiotherapy [4].

Complete surgical synovectomy remains the treatment of choice for the disease. Pathological synovial tissue should be removed as completely as possible [1, 5, 9, 10]. Arthroscopy, in comparison with open surgery, allows for better exploration of the bursa and glenohumeral cavity for anatomical characterization, is at least as effective as open synovectomy and is less aggressive [1]. We believe, therefore, to be the gold standard for treatment.

When massive arthritic changes are present, total synovectomy alone may be insufficient and should be combined with shoulder arthroplasty [8, 10]. Recurrence occurs probably due to incomplete synovial excision, with rates varying according to the affected joint. In the shoulder recurrence is rare, as well as the occurrence, and do not differ for localized or diffuse types, nor for the bone involved [8].

In this report, we present a prolonged and atypical case of PVNS in the subacromial bursal space of a teenager’s shoulder, which could be successfully identified and treated using appropriate propedeutics and arthroscopic synovectomy as a therapeutic resource.

## CONFLICT OF INTEREST STATEMENT

None declared.
